# Assessment of cardiac time intervals using high temporal resolution real-time spiral phase contrast with UNFOLD-SENSE

**DOI:** 10.1186/1532-429X-16-S1-W15

**Published:** 2014-01-16

**Authors:** Grzegorz T Kowalik, Daniel S Knight, Jennifer A Steeden, Oliver Tann, Freddy Odille, David Atkinson, Andrew Taylor, Vivek Muthurangu

**Affiliations:** 1 Institute of Cardiovascular Science, UCL Centre for Cardiovascular Imaging, London, UK; 2 Royal Free Campus, UCL Division of Medicine, London, UK; 3 Cardiorespiratory Unit, Great Ormond Street Hospital for Children, London, UK; 4 U947, INSERM, Nancy, France; 5 Université de Lorraine, Nancy, France; 6 Center for Medical Imaging, UCL Division of Medicine, London, UK

## Background

Cardiac time intervals (CTI) provide information about systolic and diastolic function and can be measured using mitral valve (MV) and LV outflow tract (LVOT) Doppler velocity curves. Velocity curves produced by gated phase-contrast (PC) MR are not suitable for measurement of CTI due to distortion secondary to averaging across multiple heartbeats. Therefore, we developed a novel real-time spiral UNFOLD-SENSE PCMR sequence with high temporal resolution necessary to measure CTI. The purpose of this study was to i) validate CTI measurement against Doppler and; ii) assess CTI during exercise.

## Methods

**Figure 1:**
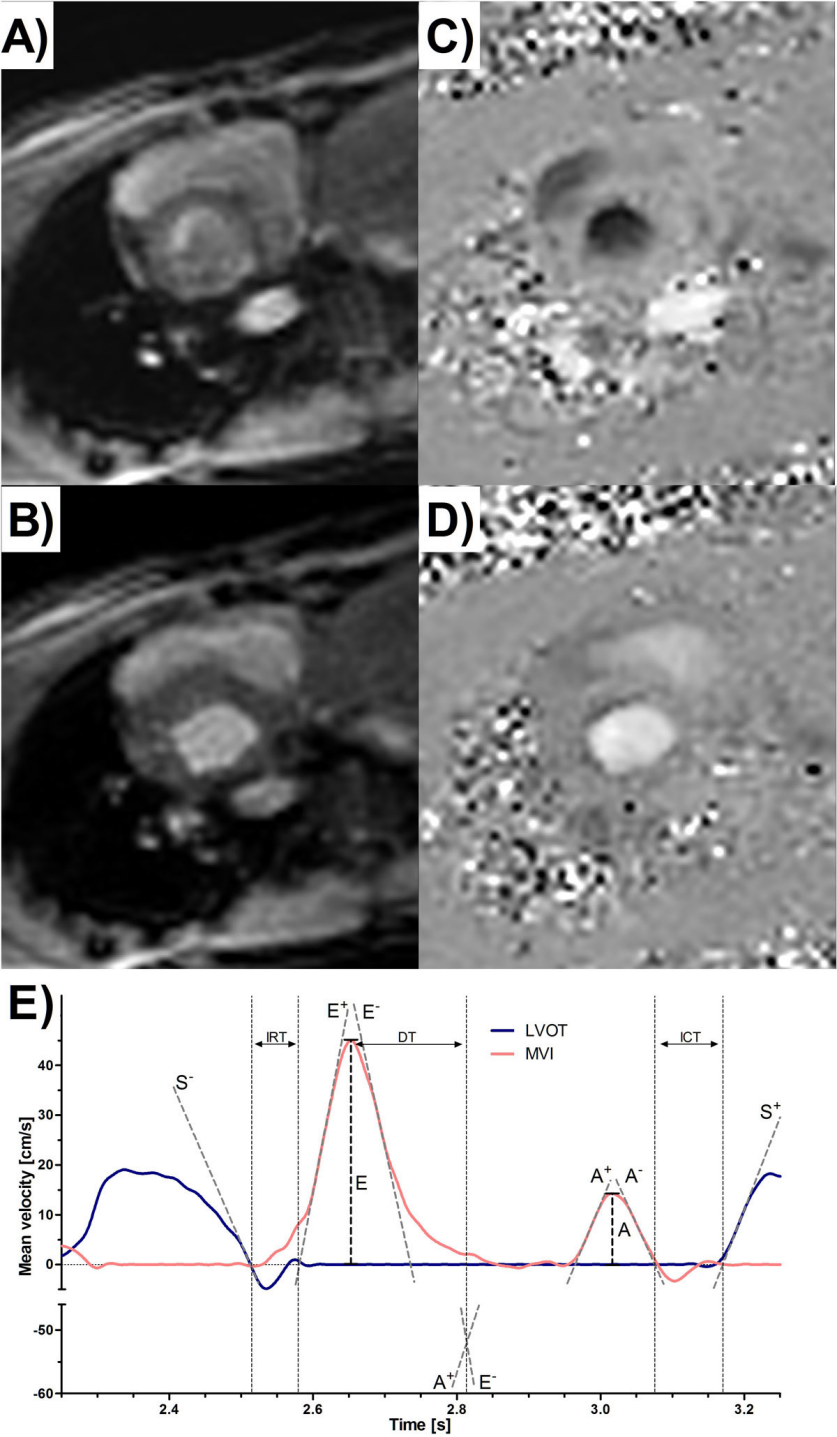
**Example of magnitude (A, B) and phase (C, D) images reconstructed with the new reconstruction and for data acquired at systole (A, C) and diastole (B, D) periods for the same subject**. E) Plot of left ventricular outflow tract (LVOT) and mitral valve inflow (MVI) velocity curves. The start and end of the S, E and A waves are delineated by the horizontal axis intercepts of tangent lines drawn on the ascending (+ superscript) and descending (- superscript) slopes of the respective waves. These are then used to calculate: IRT - isovolumic relaxation time, ICT - isovolumic contraction time and ET - ejectiontime as shown.

All subjects were imaged using uniform density spiral PCMR real-time sequence (voxel: 3.5 × 3.5 mm, TR/TE: 7.41/1.97 ms, 10 interleaves for fully sampled k-space, B0 = 1.5T). A novel interleaved acquisition was implemented to allow a combination of UNFOLD with SENSE. UNFOLD filtering (-3dB cut-off frequency at ≈25Hz) was performed on blocks of 20 frames with each frame containing a single alternating interleave (R = 10). After UNFOLD the affective acceleration was 5x. As the pair of interleaves acquired in each block was different (i.e. block 1: interleaves 1 & 6, block 2: interleaves 2 & 7), combining 5 adjacent blocks produced a fully sampled k-space used to create coils sensitivity maps. Thus, SENSE reconstruction was performed on groups of 100 frames. The resultant sequence had a temporal resolution ≈14.8 ms. 15 healthy volunteers were recruited for the validation study and underwent Doppler echo and both our real-time PCMR sequence and a conventional high temporal resolution (≈10 ms) gated PCMR. Ten volunteers were recruited for the exercise study, in which subjects were imaged prone to facilitate the alternating weighted knee flexion exercise. Mean-velocity data at the MV and LVOT were extracted and used to calculate ejection time, isovolumic contraction time, isovolumic relaxation time (Figure 1).

## Results

The protocol was successfully performed in all volunteers and the reconstruction produced good quality images (Figure 1) that allowed subsequent measurement of CTI. There was good agreement for all CTI between UNFOLD-SENSE data and Doppler. However, there was poor agreement between gated PCMR and Doppler. The exercise study showed a mean increase in heart rate of ≈30%. All CTI were significantly lower (P < 0.05) during exercise (Table 1).

**Table 1:** Combined validation (Bland-Altman and correlation analyses) and exercise (mean ± standard deviation and P values of paired two-tailed t-test) results.

		ICT	IRT	ET
Echo		46.4 ± 12.6	73.8 ± 14.7	301.1 ± 21.4

Gated		39.6 ± 12.3	56.7 ± 12.5	310.3 ± 20.9

UNFOLD-SENSE	Mean [ms]	45.0 ± 11.6	74.1 ± 9.3	306.0 ± 19.0

	Bias [ms]	-1.4 ± 5.6	0.3 ± 9.6	4.9 ± 11.7
	
	Correlation	r = 0.898	r = 0.793	r = 0.849
	
Rest		55.9 ± 21.3	66.8 ± 8.1	276.8 ± 20.1

Stress		42.6 ± 19.5	57.9 ± 9.5	264.8 ± 21.4

Paired two-tailed t-test		P = 0.0307	P = 0.0213	P = 0.0067

## Conclusions

We have shown that using a novel real-time PCMR sequence it is possible to accurately quantify CTI. Importantly, it significantly outperforms gated PCMR, as there is no averaging of the velocity curves across multiple heartbeats. Furthermore, we have shown that it is possible to measure the change in CTI in response to exercise with MR.

## Funding

GK funded by Siemens Medical Systems, DK funded by British Heart Foundation, JAS funded by British Heart Foundation, AT funded by the National Institute for Health Research, VM funded by British Heart Foundation, The work was carried out with support of the GOSH/ICH NIHR Biomedical Research Centre.

